# Detection and Molecular Characterization of 9000-Year-Old *Mycobacterium tuberculosis* from a Neolithic Settlement in the Eastern Mediterranean

**DOI:** 10.1371/journal.pone.0003426

**Published:** 2008-10-15

**Authors:** Israel Hershkovitz, Helen D. Donoghue, David E. Minnikin, Gurdyal S. Besra, Oona Y-C. Lee, Angela M. Gernaey, Ehud Galili, Vered Eshed, Charles L. Greenblatt, Eshetu Lemma, Gila Kahila Bar-Gal, Mark Spigelman

**Affiliations:** 1 Department of Anatomy and Anthropology, Sackler Faculty of Medicine, Tel-Aviv University, Tel-Aviv, Israel; 2 Centre for Infectious Diseases and International Health, University College London, London, United Kingdom; 3 School of Biosciences, University of Birmingham, Edgbaston, Birmingham, United Kingdom; 4 Biosciences Research Institute, University of Salford, Salford, United Kingdom; 5 Marine Archaeology Branch, Israel Antiquities Authority, Jerusalem, Israel; 6 Kuvin Center for the Study of Infectious and Tropical Diseases, Hebrew University-Hadassah Medical School, Jerusalem, Israel; 7 Koret School of Veterinary Medicine, Hebrew University of Jerusalem, Rehovot, Israel; Centre for DNA Fingerprinting and Diagnostics, India

## Abstract

**Background:**

*Mycobacterium tuberculosis* is the principal etiologic agent of human tuberculosis. It has no environmental reservoir and is believed to have co-evolved with its host over millennia. This is supported by skeletal evidence of the disease in early humans, and inferred from *M. tuberculosis* genomic analysis. Direct examination of ancient human remains for *M. tuberculosis* biomarkers should aid our understanding of the nature of prehistoric tuberculosis and the host/pathogen relationship.

**Methodology/Principal Findings:**

We used conventional PCR to examine bone samples with typical tuberculosis lesions from a woman and infant, who were buried together in the now submerged site of Atlit-Yam in the Eastern Mediterranean, dating from 9250-8160 years ago. Rigorous precautions were taken to prevent contamination, and independent centers were used to confirm authenticity of findings. DNA from five *M tuberculosis* genetic loci was detected and had characteristics consistent with extant genetic lineages. High performance liquid chromatography was used as an independent method of verification and it directly detected mycolic acid lipid biomarkers, specific for the *M. tuberculosis* complex.

**Conclusions/Significance:**

Human tuberculosis was confirmed by morphological and molecular methods in a population living in one of the first villages with evidence of agriculture and animal domestication. The widespread use of animals was not a source of infection but may have supported a denser human population that facilitated transmission of the tubercle bacillus. The similarity of the *M. tuberculosis* genetic signature with those of today gives support to the theory of a long-term co-existence of host and pathogen.

## Introduction

Tuberculosis is a major global cause of death and disease and around 2 billion people, about one third of the world's total population, are believed to be infected with tubercle bacilli [1]. However, only around 10% of infected persons become ill with active disease, and this high level of latent infection is an indication of long-term co-existence of human host and bacterial pathogen [2]. Tuberculosis is caused by a group of closely related bacterial species termed the *Mycobacterium tuberculosis* complex. Today the principal cause of human tuberculosis is *Mycobacterium tuberculosis*. *Mycobacterium bovis* has a wider host range and is the main cause of tuberculosis in other animal species. Humans become infected by *M. bovis*, usually via milk, milk products or meat from an infected animal. It is estimated that in the pre-antibiotic era *M. bovis* was responsible for about 6% of tuberculosis deaths in humans [3], [4]. Other members of the *M. tuberculosis* complex include the human pathogens *Mycobacterium canettii*, *Mycobacterium africanum*, and species usually associated with animal infections, such as *Mycobacterium microti*, *Mycobacterium caprae* and *Mycobacterium pinnipedii*.

Tuberculosis can cause characteristic skeletal changes, such as collapse of the vertebrae (Pott's disease), periosteal reactive lesions, and osteomyelitis [5]. Such paleopathological changes have been reported in pre-dynastic (3500-2650 BC) Egypt [6], and Neolithic (3200-2300 BC) Sweden culturally associated with the earliest cattle breeders [7]. These are the oldest cases of human tuberculosis confirmed by ancient DNA. Older cases recognized by skeletal changes alone were found in Neolithic Italy at the beginning of the fourth millennium BC [8], [9].

Erosive lesions suggestive of tuberculosis have been found on fossil fauna from the Natural Trap Cave in Wyoming, dated from the 17,000 to 20,000 year level [10] and tuberculosis in one specimen was confirmed by biomolecular methods [11]. Initially it was believed that humans acquired tuberculosis from animals, especially after domestication [12]–[14]. Whole genome sequencing has since revealed that the *M. tuberculosis* complex has accumulated deletions over time, which can be used to distinguish individual species and lineages [15] and earlier ideas about the evolution of the *M. tuberculosis* complex have been revised [16], [17]. An intriguing indication of the antiquity of the disease is the finding of non-specific morphological changes consistent with tuberculosis in a fossil *Homo erectus* dating from the middle Pleistocene (490–510,000 years BP) from Turkey [18].

The emergence of human infectious diseases has been linked to changes in human ecology and to interactions between populations [19]. The change from a gatherer-hunter lifestyle to settled farming communities appears to coincide with the appearance of diseases such as smallpox, measles, malaria, schistosomiasis, and tuberculosis [20]. Our aim was to investigate this stage of human history by the use of molecular methods to examine human remains that pre-date the earliest verified cases of tuberculosis, but with paleopathology consistent with this disease. A further aim was to elucidate the molecular characteristics of the causative organism. It is believed that the denser, settled populations associated with agriculture and animal domestication enabled human pathogens such as *M. tuberculosis* to be maintained indefinitely [21]. Therefore, we examined one of the earliest villages with evidence of both animal domestication and agriculture, Atlit-Yam [22], for the presence of tuberculosis in human remains with characteristic lesions. *M. tuberculosis* was confirmed in the skeletal remains of a woman and child, using both ancient DNA and bacterial cell-wall specific lipid markers. Deletion analysis indicates that the modern *M. tuberculosis* lineage characterized by the TbD1 deletion existed 9000 years ago.

## Materials and Methods

**Figure 1:**
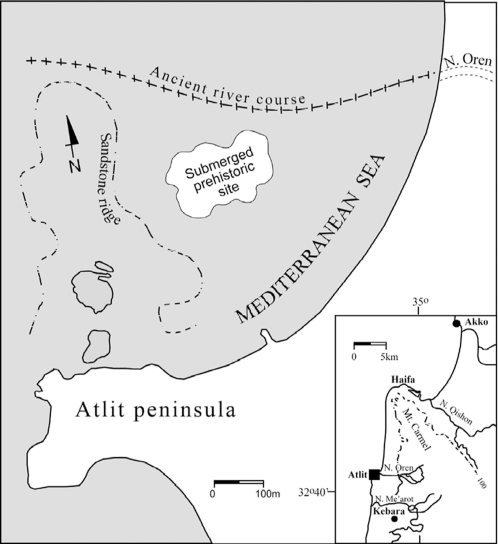
Map of Atlit-Yam site in the North Bay of Atlit, 10 km south of Haifa (34°56′ E, 32°42.5′ N). Inset shows general geographical location.

The site of Atlit-Yam is now located 300–500 m offshore, (34°56′ E, 32°42.5′ N), 8–12 m below sea level in the North Bay of Atlit, 10 km south of Haifa (Figure 1). Calibrated radiocarbon dates range from 9250-8160 years BP [23], indicating a date during the last phase of the Pre-Pottery Neolithic C period, when human society accomplished a full shift from hunting and gathering to farming, fishing and animal husbandry. The rich finds included botanical remains, tools, animal and human bones. The many animal remains that were excavated included goat (44%), cattle (43%), pig (9%), gazelle and deer (3.3%).

Human skeletons, which were embedded in dark clay, were carefully excavated and soaked in fresh-water tanks to dissolve the salts. The skeletal remains [24], [25] were generally well-preserved (Supporting Figure S1A) and some showed paleopathological features consistent with a diagnosis of tuberculosis. Samples were taken for molecular examination from the skeletal remains of a woman buried together with an infant (Supporting Figure S1B). We analyzed the ribs, arm bones (adult) and long bones (infant). The work was done in separate centers to provide verification of data, and stringent precautions were taken against contamination (Supporting Materials and Methods S1). Separate areas and pipettes were used for extraction, PCR set-up and product analysis. Filter tips were used routinely and surfaces and equipment were cleaned before each assay. DNA extracts were prepared and, using the polymerase chain reaction (PCR), both multi-copy and single copy target loci were amplified (Table 1) and sequenced to confirm their identity. Screening PCRs detected the *M. tuberculosis* complex and nested or hemi-nested PCR was used to increase the likelihood of detection (Supporting Materiasls and Methods S1). A single copy conserved membrane protein locus (CMP) found in the *M. tuberculosis* complex was examined by PCR to assess the feasibility of seeking further single-copy loci. PCR target regions, based on specific deletions, were used to distinguish between *M. tuberculosis* and *M. bovis*. Extracts were analyzed further by reverse dot-blot hybridization of the *M. tuberculosis* complex-specific Direct Repeat (DR) region, a procedure known as spoligotyping [26]. In addition, samples from both the infant and adult were analyzed by high performance liquid chromatography (HPLC) for mycobacterial cell wall mycolic acids [27], [28] (Supporting Tables S1, S2 and S3 and Supporting Figure S2 A and B). Long chain fatty acids were converted to pyrenebutyric acid-pentafluorobenzyl mycolates, and reverse phase HPLC examined for profiles similar to standard *M. tuberculosis*. Further normal and reverse phase HPLC was performed to give detailed profiles for each sample. These were used to determine the percentage ratios and absolute amounts of mycolic acids extracted from bone samples.

**Table 1:** Primer sequences and PCR details.^1^

Locus	Primers (5′ - 3′)	MgCl_2_ (mM)	Annealing temp. (°C)	Product (bp)
IS*6110*	P1: CTCGTCCAGCGCCGCTTCGG			
Outer	P2: CCTGCGAGCGTAGGCGTCGG	1.5	68	123
IS*6110*	IS3: TTCGGACCACCAGCACCTAA			
Nested	IS4: TCGGTGACAAAGGCCACGTA	1.5	58	92
IS*1081*	F2: CTGCTCTCGACGTTCATCGCCG			
Outer	R2: GGCACGGGTGTCGAAATCACG	1.5	58	135
IS*1081*	F2: CTGCTCTCGACGTTCATCGCCG			
Hemi-nested	R3: TGGCGGTAGCCGTTGCGC	2.0	58	113
TbD1	TbD1a: CTAACGGGTGCAGGGGATTTC			
Flanking outer	TbD1b: CCAAGGTTACGGTCACGCTGGC	1.5	60	128
TbD1	TbD1c: GCAGGGGATTTCAGTGACTG			
Flanking inner	TbD1d: GCTGGCCAGCTGCTCGCCG	1.5	58	103
CMP	F2: TCGGTCAGCAAGACGTTGAAG			
	R: ACTTCAGTGCTGGTTCGTGG	2.0	58	105
RD2	BV1: ATCTTGCGGCCCAATGAATC			
Outer	BV2: CAACGTCTTGCTGACCGACA	1.5	58	124
RD2	BV3: ATGAATCGGCCGCGTTCG			
Nested	BV4: GACCGACATCGGTGCCGCG	1.5	58	99
DR	DRa: GGTTTTGGGTGTGACGAC 2			Not applicable
	DRb: CCGAGAGGGGACGGAAAC	3.0	55	

1 An initial denaturation step (95°C for 15 mins – hot start PCR, or 94°C for 1 min); DNA amplification (initially 40 cycles, with 25 cycles in nested reactions) of strand separation at 94°C for 40 sec, 1 min of primer annealing, followed by strand extension at 72°C for 20 sec plus 1 sec/cycle; and a final extension step at 72°C, were used for all PCR amplifications.

2 The DRa primer was biotinylated at the 5′ end to enable subsequent detection of amplified DNA by reverse hybridization.

## Results

### Paleopathology

**Figure 2:**
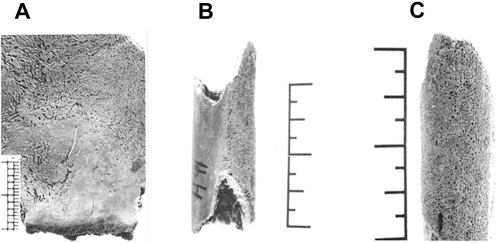
Paleopathological lesions on Neolithic infant bones. A. Endocranial surface of the infant showing marked engravings (*serpens endocrania symmetrica*, SES), which indicate chronic respiratory malfunction, and are usually associated with tuberculosis. B. Fragment of long bone of the infant. Note the intensive bone remodeling (hypertrophic osteoarthropathy, HOA) at the surface on the right side. C. Higher magnification of the HOA on the infant bone.

The infant, though small in size, was estimated to be about 12 months old, based on crown development and long bone dimensions. On the inner aspect of the infant cranial bones were serpentine engravings (*serpens endocrania symmetrica*, SES; Figure 2A), a reliable diagnostic criterion for intra-thoracic inflammation [29] and associated with tuberculosis. The infant tubular bones also demonstrated lesions identified as hypertrophic osteoarthropathy (HOA), highly suggestive of tuberculosis [30] and characterized by the formation of an expanded shell of periosteal reactive bone (Figure 2B and C). The woman was estimated to be around 25 years old, based on dental attrition, epiphyseal ring ankylosis and symphysis pubis. There was a slight periosteal reaction affecting the distal diaphysis of the one tibia available for examination, a bony change consistent with HOA [5], [31], [32]. However, the changes were not so marked as to be diagnostic.

### *Mycobacterium tuberculosis* complex and *M. tuberculosis* DNA

*M. tuberculosis* complex DNA was detected in the bones of woman and infant (Table 2). Positive results with the multi-copy IS*6110* [33], [34] and IS*1081* [35] PCRs were obtained with the rib sample from the woman and infant long bone sample, and confirmed by sequencing. An IS*6110* 123 bp product from the woman (right rib) and a 92 bp nested IS*6110* product from the infant were obtained, identical to those in the NCBI database. Additionally, a 104 bp sequence identical to the relevant NCBI sequence in the IS*1081* product was obtained from the infant.

**Table 2:** Summary of PCR results.

PCR locus	Woman		Infant	
	1st stage PCR	2nd stage PCR	1st stage PCR	2nd stage PCR
IS*6110*	Positive	Positive	Positive^1^	Negative
IS*1081*	Positive	Positive	Positive^1^	Positive
Flanking TbD1	Positive	Negative	Positive^1^	Positive
RD2	Negative	Negative	Negative	Negative
CMP	Positive	Not done	Positive^1^	Not done

1 confirmed by sequencing.

The single copy TbD1 flanking PCR was positive from the infant sample and a complete DNA sequence for the 128 bp amplicon with the outer primers was obtained (Supporting Figure S3A and B). The consensus sequence was identical to that in the NCBI database. The strong signal indicates the excellent preservation at this locus of the *M. tuberculosis* DNA template. Nested PCR was also successful. Weak positives were obtained with the outer primers from the female sample. These findings are evidence that the infecting organism was *M. tuberculosis* from a lineage in which the TbD1 deletion had occurred [16]. Results from the infant for the single copy CMP PCR were faint and a partial sequence was obtained (Supporting Figure S3C) with some mismatched bases compared with the database, attributed to poor DNA preservation. No demonstrable amplicons of human nuclear microsatellite DNA were obtained from the bone samples. Spoligotyping provided additional evidence for *M. tuberculosis* complex DNA for both the adult and infant specimens (Supporting Figure S4 A–D), although there were several faint or dubious positives and inconsistencies between replicates, as might be expected of ancient specimens.

### Lipid biomarkers of *M. tuberculosis*

**Figure 3:**
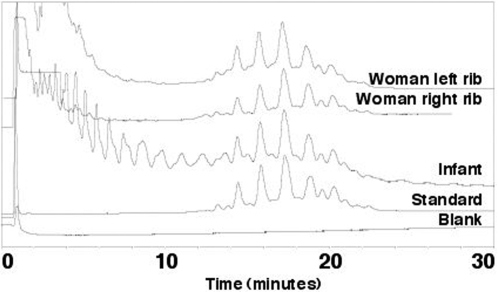
Detection of *Mycobacterium tuberculosis* mycolic acid pyrenebutyric acid-pentafluorobenzyl (PBA-PFB) derivatives by reverse phase fluorescence high performance liquid chromatography (HPLC), from the Neolithic woman and infant. The characteristic tight envelopes of peaks are the total mixture of homologues for the different α-, methoxy- and ketomycolates. The Y-axis in the profiles represents absorbance; absolute values of the mycolates detected are shown in Supporting Table 5.

Modifying an established protocol [28], [36], long-chain fatty acids were extracted as pentafluorobenzyl (PFB) esters, and fractions corresponding to PFB mycolates were obtained (Supporting Tables S4, S5). After treatment with pyrenebutyric acid (PBA) these fractions produced PBA-PFB mycolates, which, after reverse phase HPLC, gave profiles closely similar to those produced by the *M. tuberculosis* complex, as indicated by a standard *M. tuberculosis* strain (Figure 3). Further normal and reverse phase HPLC gave detailed profiles for each sample, reinforcing the close identity with *M. tuberculosis* (Supporting Figure S2 C and D).

## Discussion

It is believed that inundation of the Atlit-Yam site occurred shortly after abandonment [24] and thereafter the environment remained unchanged for 9,000 years. The Atlit-Yam site was located within marshland; the graves were encased in clay, eventually covered by thick layer of sand and later by salt water, thus providing anaerobic conditions that retard degradation. The excellent preservation of the skeletal remains is consistent with the excellent physical state of the organic artifacts (wooden bowls, reed mats) that were found on the site. The paleopathogical lesions of SES in the infant cranium and HOA in the infant long bones and possibly also the tibia of the woman buried with the infant, presumed to be the mother, suggest that both suffered from, and died of, tuberculosis.

Anaerobic conditions are also conducive for DNA preservation [37] and DNA analysis supports the paleopathological diagnosis of tuberculosis. Overall, the PCR data provide strong evidence of the *M. tuberculosis* complex as specific DNA was detected in five different genetic loci, including the TbD1 locus with a deletion that is specific for a broadly-defined modern lineage of *M. tuberculosis*. Failure to detect human DNA (data not included) may reflect the greater stability of the GC-rich mycobacterial DNA, which additionally benefits from the robust hydrophobic bacterial cell wall [28], [38], [39].

Direct detection of cell wall mycolic acids specific for the *Mycobacterium tuberculosis* complex, without any amplification step, provides independent, robust confirmation of the presence of tuberculosis. The quantity of mycolic acids appeared lower in the infant sample (Supporting Table S5), in contrast to the DNA studies where the infant gave better results. However, the mycolate analyses were carried out on three combined rib samples from the baby, not all of which had been tested for MTB DNA. These extremely hydrophobic high molecular weight molecules are more stable than DNA and have been used previously to confirm diagnoses of ancient tuberculosis [27], [28].

We conclude that both individuals in our study were infected with *M. tuberculosis*, and that our findings are supported when we consider the nature of the site, the stringent precautions taken to prevent cross-contamination and verification by the specific lipid biomarkers. Furthermore, we believe that this is the earliest report of the disease in humans that has been confirmed by molecular means. The infant is likely to have had disseminated primary tuberculosis: - the only DNA sequences for single copy loci were obtained from the infant material, which suggests a higher bacterial load during life. In infants less than a year old the present risk of developing active disease on infection with *M. tuberculosis* is as high as 43% [40] due to the inadequacy of their immune system. This compares with 5–10% in adults, 15% in adolescents, and 24% in children aged 1–5 years.

The size of the infant's bones, and the extent of the bony changes, suggest a case of acquired neonatal tuberculosis, in which an adult suffering from contagious pulmonary tuberculosis infects an infant shortly after birth. Childhood tuberculosis is closely linked to adult disease and is usually a sentinel event in the community, demonstrating recent transmission. Infant and maternal mortality rates from untreated tuberculosis in recent times was between 30% and 40% [41], so it is unsurprising for both mother and child to succumb and be buried together.

Spoligotyping should be a useful method of examining DNA from archaeological material as even fragmented DNA gives results, due to the increased sensitivity from the combination of amplification and hybridization [42]. *M. tuberculosis* complex DNA from the lesion of a 17,000-old extinct Pleistocene bison [11] yielded spoligotyping patterns most similar to *Mycobacterium africanum* or *M. tuberculosis* [43], and distinct from present day *M. bovis*. Zink *et al.* [44] obtained spoligotypes from ancient Egyptian human bone and soft tissue samples, dating back to about 4000 years. Of their 12 positive samples, spoligotyping indicated *M. tuberculosis* or, in some older Middle Kingdom samples, *M. africanum* patterns, but not those of *M. bovis*.

We carried out spoligotyping on specimens from the woman and infant but replicated typing gave inconsistent results, which suggests there may be poor DNA preservation of some of the single-copy spacer regions. The observed patterns do not match any in the International Data Base spoldb4: www.pasteur-guadeloupe.fr/tb/spoldb4 but appear similar to an ancestral pattern. Results need to be interpreted with caution, as spoldb4 is based on data obtained from cultured organisms and spoligotyping has not been validated for application to DNA extracts prepared from degraded or archival specimens. The spoligotyping technique is based on modern *M. tuberculosis* strains from around the world. As the main variation in types is caused by unidirectional deletions, all ancestral strains are likely to produce a near-complete profile of the DR region and therefore to resemble each other. This may explain why the spoligotypes from the Atlit Yam skeletons resemble those of the Pleistocene bison [11].

Deletion analysis is a more robust method of examining ancient material [16], [45], and based on the TbD1 deletion, the genetic lineage resembles modern lineages of *M. tuberculosis* [16], [43], [51].

Suggestions that human tuberculosis arose from *M. bovis* in hunted or domesticated animals have been revised since comparative genomic studies demonstrate that *M. bovis* represents a later lineage [16], [17]. Members of the *M. tuberculosis* complex are genetically very similar and were believed to be the result of a clonal expansion following an evolutionary bottleneck 20,000–35,000 years ago [16], [46], [47]. However, further genomic studies of the *M. tuberculosis* complex indicate a more ancient origin of this group of closely related species than had previously been believed, and that possibly an early progenitor, perhaps similar to *M. canettii*, was present in East Africa as early as 3 million years ago [48], [49]. The observation of non-specific lesions consistent with tuberculosis found in a 500,000 year-old skeleton of *Homo erectus* [18] may also indicate the long-term co-existence of host and pathogen, although the diagnosis in this particular case has been questioned. However, *M. tuberculosis* appears to have undergone long-term co-evolution with its human host prior to the evolutionary bottleneck and well before the development of agriculture and domestication, comparable to other long-term human pathogens such as *Helicobacter pylori* [50], [51].

The present study of a population from 9250-8160 years ago, around the time of the first great transition from hunter-gatherers to a settled agriculture-based lifestyle [19], helps us to understand the nature of tuberculosis within the Middle East. Could the presence of cattle be pertinent? Atlit-Yam is among the very few Pre-Pottery Neolithic sites where domesticated cattle have been found. Furthermore, it is the only Neolithic site where there were quantities of bovine bones, indicating that cattle were a major dietary component [23]. We suggest that in the absence of detectable *M. bovis*, the cattle may be important by supporting a larger and denser human population, thus indirectly encouraging the conditions for the long-term maintenance and transmission of *M. tuberculosis* [21].

## Supporting Information

Materials and Methods S1 Text
(0.05 MB DOC)

Table S1 The solvent sequence used for the silica gel normal phase cartridge fractionation of long-chain compounds
(0.03 MB DOC)

Table S2 The solvent sequence used for the reverse phase cartridge purification of PBA-PFB mycolates
(0.02 MB DOC)

Table S3 Conditions for HPLC analysis of PBA-PFB mycolates
(0.02 MB DOC)

Table S4 Percentage ratios of alpha-, methoxy- and ketomycolates in archaeological samples and *M. tuberculosis* standard determined in normal phase HPLC
(0.02 MB DOC)

Table S5 Absolute amounts of mycolic acids extracted from bone samples
(0.02 MB DOC)

Figure S1 Atlit-Yam burials. A. An example of human remains with excellent preservation. B. Partial excavation of the burial site with the adult female and infant skeleton (arrow).
(2.46 MB TIF)

Figure S2 High Performance Liquid Chromatography (HPLC) methodology. A. Representative structures of the mycolic acids from *M. tuberculosis*. Natural mixtures of mycolates express a range of homologous components with varying chain lengths. B. Strategy for the release and derivatization of mycolic acids for fluorescence HPLC. (a) Hydrolysis with KOH/ methanol/toluene to release mycolic acids. (b) Phase-transfer catalyzed esterification of mycolic acids with pentafluorobenzyl bromide (PFB) to give PFB mycolates. (c) Esterification of PFB mycolates, by reaction with pyrenebutyric acid (PBA), to produce PBA-PFB mycolates, catalyzed by dicyclohexylcarbodiimide and pyrrolidinopyridine. R- represents the remainder of the mycolate molecule. C. Normal phase HPLC of PBA-PFB mycolates from bone samples and standard *M. tuberculosis*. D. Reverse phase HPLC of individual α -, methoxy- and ketomycolic acid PBA-PFB derivatives from bone samples and standard *M. tuberculosis*. The number of carbons in the individual underivatized mycolic acids is shown.
(0.83 MB TIF)

Figure S3 DNA sequence data. A. *M. tuberculosis* TbD1 flanking region (128 bp), obtained from the infant sample (5′-3′ strand). B. *M. tuberculosis* TbD1 flanking region (128 bp), obtained from the infant sample (3′-5′ strand). C. *M. tuberculosis* conserved membrane protein (CMP) region, obtained from the infant sample (5′-3′ strand only).
(12.09 MB TIF)

Figure S4 Repeated spoligotypes from the Atlit-Yam samples and controls. Each set of spoligotyping data (A–C) represents, from top to bottom, *M. tuberculosis*, *M. bovis* (BCG) controls, Atlit Yam female and Atlit Yam infant. D. Diagram of spoligotyping data, including *M. africanum* spoligotypes (Donoghue et al 2004), and a consensus pattern based on one or more positive results.
(5.61 MB TIF)
