# Supplementary material for: Detection and Molecular Characterization of 9000-Year-Old Mycobacterium tuberculosis from a Neolithic Settlement in the Eastern Mediterranean
Source: PLoS One. 2008 Oct 15;3(10):e3426. doi: 10.1371/journal.pone.0003426 (PMC2565837; doi:10.1371/journal.pone.0003426)
Supplement: Materials and Methods S1 — Text (0.05 MB DOC) [file pone.0003426.s001.doc]

# Supporting Materials and Methods S1

## Molecular Materials and Methods

### Samples

Bone samples were taken on different days using stringent standard precautions [1], with protective clothing and sterile instruments [2]. Material was taken directly to each of the laboratories after sampling in Tel Aviv.

### DNA extraction

Three separate centers were used: -

Center 1: UCL, London;

Center 2: School of Veterinary Medicine, The Hebrew University of Jerusalem, Rehovot;

Center 3: Kuvin Center for the Study of Infectious and Tropical Diseases, The Hebrew University, Hadassah Medical School, Jerusalem.

All centers took stringent precautions against cross-contamination with separate rooms for different stages of the process. The procedures have been described previously [2]. The strategy emphasizes physical barriers with separate areas for extraction, PCR set-up and product analysis. Separate sets of pipettes were used for PCR set-up and product analysis. The former was stripped and cleaned in detergent and ethanol before each experiment. Filter tips were used routinely. Surfaces and equipment in contact with sample tubes (centrifuges, rotors, mixers, etc.) were cleaned before each assay. In center 2, which also examined samples for human DNA, DNA extractions and PCR setups took place in separate ultraviolet-exposed hoods to prevent contamination by contemporary DNA. In addition, all reagents and tubes were irradiated with ultraviolet light. In each center multiple sample blanks were used for negative controls during the DNA extraction and water blanks were included in PCR amplifications to ensure there was no contamination.

Initially, DNA extraction was carried out according to the protocols described previously [2] based on initial demineralization, guanidium thiocyanate-based lysis buffer [3] and capture onto silica [4]. Thereafter, in center 1 the protocol was modified [5] and DNA was extracted using NucliSens® reagents (bioMérieux UK Ltd, Hampshire, UK). Crushed bone sample (25-120 mg) was added to 9ml tubes of lysis buffer, mixed on a rotator for 30 mins and boiled for at least 5 mins. Next, samples were frozen in liquid nitrogen and thawed for three cycles. Silica capture of DNA and washing of the silica was according to the manufacturer's instructions. DNA was eluted in 60 µl TE buffer and used immediately, if possible. Extracts were stored at -20^o^C. In center 2 DNA from both female and child was extracted independently three times. From center 2, DNA was aliquoted and used for both PCR and spoligotyping.

### DNA amplification to detect *M. tuberculosis*-complex (MTB) specific DNA

DNA extracts were screened for the presence of *M. tuberculosis* DNA by PCR (Table 1). Two specific regions found in all members of the *M. tuberculosis* complex, based on the insertion elements IS*6110* [6,7] and IS*1081* [8], which are normally multi-copy, were used to increase the likelihood of detection. In addition, nested or hemi-nested PCR, respectively, was used. A short sequence found in a region believed to code for a conserved membrane protein (CMP) was used to assess the feasibility of detecting a single site locus. Additional PCR target regions, based on specific deletions, were used to distinguish between *Mycobacterium tuberculosis* and *Mycobacterium bovis*. Flanking primers were used to detect the TbD1 deletion, specific for non-ancestral lineages of *M. tuberculosis* complex [9], and a deletion adjacent to the RD2 deletion locus [10], found in *M.bovis* [11].

PCR was carried out in 25 µl volumes using 5 µl of DNA extract, according to the parameters in Table1. Negative controls containing water were always run in parallel. Qiagen Hotstar® *Taq* polymerase and reagents (Qiagen, West Sussex, UK) were used for first stage PCR reactions. Bovine serum albumin (BSA) at a final concentration of 10 mM was included in one set of reactions, as this has been shown to improve PCR yield [12,13]. Nested or hemi-nested PCR was performed using pre-aliquoted double-strength PCR mix (ABGene®, Surrey, UK). The final composition of the PCR mixture (50 µl) was 75 mM Tris/HCl (pH 8.8); 20 mM (NH_4_)_2_SO_4_; 1.5 mM MgCl_2_; 0.01% (v/v) Tween 20; 200 µM (each) dATP, dCTP, dGTP and dTTP; and 1.25 units *Taq* DNA polymerase. The primer pair and DNA preparation (1.0 µl) were added to each pre-aliquoted tube plus sufficient water to bring the volume to 50µl. Primer concentrations were between 125-500nM.

In centers 2 and 3 the same specific oligonucleotide primers for IS*6110* were used on the DNA extracts made locally to ensure independent verification. PCR amplification was conducted using high-fidelity Taq-Gold (ABI) to minimize polymerase error. Two pairs of primers were used to amplify segments of the mitochondrial ribosomal gene. Touchdown PCR was performed in a volume of 25 µl with initial denaturation at 95°C for 10 min followed by a total of 45 cycles of 15 s at 94°C, 30 s annealing for 2 cycles each at 60°C, 58°C, 56°C, 54°C, 52°C, and 35 cycles at 50°C or 48°C, and 45 s elongation at 72°C, with a final extension of 10 min at 72°C. PCR.

### Detection and sequencing

In center 1 PCR product (7 µl) was added to 3 µl loading buffer (Sigma) and electrophoresed in a 3.0%(w/v) NuSieve 3:1 agarose gel (FMC Bioproducts, Flowgen) in TBE buffer (0.09 mol^-1^ Tris-borate and 0.002 mol^-1^EDTA) at 8.8 volts cm^1^ for 80 min. Amplified DNA was visualized by ethidium bromide staining exposed under ultraviolet light and was recorded with a Polaroid camera.

When sequencing was undertaken, PCR amplicons were separated on 1-2% agarose gels in TAE buffer. Bands were excised under ultraviolet light with single-use sterile scalpel blades, prepared and sequenced, using a commercial DNA purification kit and sequencing service (Supporting Figure S3 A-C). In centers 2 and 3 positive amplicons were purified and sequenced on ABI 3700 and 3730 automated sequencers (ABI) using BigDye terminator chemistry (ABI).

### Spoligotyping

This was carried out in center 3. The procedures followed were exactly as recommended in the manufacturers' manual (Isogen Life Science) except that 5% dimethylesulfoxide was included in the PCR mix and the number of amplification cycles was increased to 45. Briefly, PCR was performed using primers DRa and DRb with a biotin-label on the latter. The PCR products were hybridized onto a commercial membrane prepared with 43 spacer oligonucleotides. After hybridization, the presence of each spacer region is shown by the presence of the biotin label at the appropriate site on the membrane (Supporting Figure S4 A-D). Each PCR and spoligotyping manipulation was performed in three rounds of experiments for the test DNA samples and for the control DNA of *M tuberculosis* and *M bovis* BCG that were provided with the kit. All work with the control samples was kept separate both geographically and temporally. The spoligotyping result were read using an automated image reader (LAS 3000, FUJIFILM) with an incremental exposure time of 30 seconds until optimal patterns were observed on the screen. Spoligotyping patterns were interpreted by comparing the result with the International Data Base spoldb4: [www.pasteur-guadeloupe.fr/tb/spoldb4](http://www.pasteur-guadeloupe.fr/tb/spoldb4) and visual examination for the presence or absence of specific spacers relevant for the identification of *M. bovis* [14], *Mycobacterium africanum* [15] and modern tubercle bacilli.

### Examination for human DNA

In center 2 the initial extract was used in an attempt to genotype the human DNA using the ABI human forensic markers, to determine any relationship between the putative mother and child. However, no demonstrable amplicons of nuclear microsatellites were obtained from bone samples. This work was not pursued; neither were mitochondrial DNA markers sought, due to the possibility of external contamination during excavation.

## Lipid Biomarker Materials and Methods

### Biomarkers and derivatisation strategy

Representative structures of the mycolic acids are shown in Supporting Figure S2A; the overall derivatization strategy is shown in Supporting Figure S2B.

### Hydrolysis of bone and standard *M. tuberculosis*

Portions of right (483mg) and left (635mg) ribs from the adult skeleton and three rib fragments, combined together (589mg), from the child were crushed in clean pestles and mortars. These samples, a sample of freeze-dried M. tuberculosis PN ­– a standard tuberculin production strain, supplied by the Central Veterinary Laboratory, Weybridge, UK (5mg) and a reagent-only blank were heated overnight at 100^o^C, in separate 8.5ml PTFE capped tubes, with 30% KOH in methanol (2ml) and toluene (1ml). In an ice bath, the mixture was acidified to pH1 with 10% aqueous HCl (1ml) and drop wise addition of concentrated HCl. Toluene (1ml) was added, the solution mixed, and the toluene layer transferred to a new 8.5ml PTFE capped tube; the bottom aqueous layer was re-extracted twice with toluene (1ml), the combined toluene extracts washed with distilled water (2ml) and evaporated to dryness under a stream of air at 37^o^C. The resulting long-chain compounds were treated with phase transfer catalyst solution (2ml), {(NaOH [0.8g] and tetra-n-butylammonium hydrogen sulfate (TBAH.SO_4_) [3.39g] in water [100 ml]} and 1% pentafluorobenzyl bromide in dichloromethane (2ml) followed by mixing on a tube rotator for 1 h. In an ice bath, 10% aq. HCl (2ml) was added to the sample and the upper aqueous layer was transferred to a new tube, where it was washed thrice with toluene (1 ml). The lower organic layer was washed with distilled water (1ml), added to the combined toluene extracts and the solution evaporated to dryness at 37^o^C. The composition of the extract from standard cells was checked by TLC (heptane/ethyl acetate, 9:1).

### Purification of long-chain compounds.

Long-chain compounds were dissolved in minimal diethyl ether (~0.25ml) and loaded onto a 500mg/4ml Alltech 209250 normal phase silica gel cartridge drop wise; the tubes were rinsed with fresh diethyl ether (~0.1ml), which was also added to the silica gel cartridge. After allowing the cartridge to dry completely (~30 min or more), fractionation was carried out by use of a Supelco 57080-U Visi-1 Single SPE Tube Processor; the solvent sequence used for the fractionation is given in Supporting Table 1. Collected fractions were dried under compressed air and the extract from the standard cells was examined by TLC (heptane/ethyl acetate, 9:1).

### Preparation and purification of pyrenebutyric acid esters of PFB mycolates

The fractions from the cartridge separation, expected by reference to the standard cell extract to contain the PFB mycolates, were combined in a 4.5ml PTFE capped tube and dried thoroughly. A 2% solution (1ml) of pyrenebutyric acid in dry tetrahydrofuran was added to the tube, followed by a 1% dichloromethane solution (0.5ml) of pyrrolidinopyridine (5mg) and a 0.1M dichloromethane solution (0.5ml) of dicyclohexylcarbodiimide. The reaction mixture was stirred overnight at room temperature, quenched with distilled water (0.1ml) and toluene (0.1ml) and dried under compressed air at room temperature. The residue was partitioned between heptane (2ml) and acetonitrile (2ml), the upper heptane layer was removed to a new tube and the lower acetonitrile layer washed twice with heptane (2ml); the combined heptane extracts were dried under compressed air at room temperature. The products from the standard samples were examined by TLC (heptane/ethyl acetate, 9:1).

The dried PBA-PFB mycolate fractions, after partition, were dissolved in minimal diethyl ether (~0.25ml) and loaded on 500mg/4ml Alltech 205250 C18 reverse phase cartridges drop wise; the tubes were rinsed with fresh diethyl ether (~0.1ml), which was also added to the silica gel cartridge. After allowing the cartridge to dry completely (~30 mins or more), fractionation was carried out with a Supelco 57080-U Visi-1 Single SPE Tube Processor; the solvent sequence used is given in Supporting Table 2.

After drying with compressed air, all the fractions from the standard cell preparation were examined by TLC (heptane/ethyl acetate, 9:1). The fractions indicated to contain PBA-PFB mycolates were combined and partitioned between heptane (0.5ml) and acetonitrile (0.5ml). The upper heptane layer was removed and the lower acetonitrile layer was re-extracted twice with heptane (0.5ml); the combined heptane layers were evaporated to dryness.

### High Performance liquid chromatography (HPLC)

HPLC analyses used a VWR Hitachi Elite Pump L-2130, LaChrom Autosampler L-2200, Column Oven L-2300, operating with an IF2 User detector and Foxy Jr. Fraction Collector; the profiles were detected by Fluorescence detector L-2480 and the data processed by Scientific Software Inc. EZChrom Elite, version 3.1.6 software. Reverse phase (Alltech 81412 Alltima C18, 3μm 50 x 4.6mm) and normal phase columns (Alltech 81414 Alltima Silica, 3μm 50 x 4.6mm) were used. HPLC analysis conditions are shown in Supporting Table 3. Samples were dissolved in HPLC grade heptane (20µl -1ml) and 0.1 to 20µl was injected. Solvent blanks were run after any positive sample to ensure that there was no carry-over of material. Purified derivatives were analyzed first by reverse phase HPLC (Figure 3) and any components, corresponding to PBA-PFB mycolates, were collected and evaporated to dryness. The collected PBA-PFB mycolates were then separated into α-, methoxy- and ketomycolates by normal phase HPLC (Supporting Figure S2C); the proportions of the individual types are shown in Supporting Table 4. The fractions from the normal phase separation were dried down and re-examined by reverse phase HPLC (Supporting Figure S2D). The absolute amounts of mycolates, detected in the first reverse phase analyses, were quantified by reference to calibration curves, prepared from standard PBA-PFB mycolates (Supporting Table 5).

## References

1. O’Rourke DH, Hayes MG, Carlyle SW (2000) Ancient DNA studies in physical anthropology. Ann Rev Anthropol 29: 217-242.
2. Spigelman M, Matheson C, Lev M, Greenblatt C. Donoghue HD (2002) Confirmation of the presence of *Mycobacterium tuberculosis* complex-specific DNA in three archaeological specimens. Int J Osteoarchaeol 12: 393-401.
3. Boom R, Sol CJA, Salimans MMM, Jansen CL, Wertheim-van Dillen PME et al. (1990) Rapid and simple method for purification of nucleic acids. J Clin Microbiol 28: 495-503.
4. Höss M, Pääbo S (1993) DNA extraction from Pleistocene bones by a silica-based purification method. Nucleic Acids Res 21: 3913-3914.
5. Taylor GM, Young DB, Mays SA (2005) Genotypic analysis of the earliest known prehistoric case of tuberculosis in Britain. J Clin Microbiol 43: 2236-2240.
6. Eisenach KD, Cave MD, Bates JH, Crawford JT (1990) Polymerase chain reaction amplification of a repetitive DNA sequence specific for *Mycobacterium tuberculosis*. J Infect Dis 161: 977-981.
7. Taylor GM, Crossey M, Saldanha J, Waldron T (1996) *Mycobacterium tuberculosis* identified in mediaeval human skeletal remains using polymerase chain reaction. J Archaeol Sci 23: 789-798.
8. Taylor GM, Stewart GR, Cooke M, Chaplin S, Ladva S et al. (2003) Koch’s Bacillus – a look at the first isolate of *Mycobacterium tuberculosis* from a modern perspective. Microbiology 149: 3213-3220.
9. Brosch R, Gordon SV, Marmiesse M, Brodin P, Buchreiser C et al. (2002) A new evolutionary scenario for the *Mycobacterium tuberculosis* complex. Proc Natl Acad Sci USA 99: 3684-3689.
10. Gordon SV, Brosch R, Billault A, Garnier T, Eiglmeier K et al. (1999) Identification of variable regions in the genomes of tubercle bacilli using bacterial artificial chromosome arrays. Mol Microbiol 32: 643-655.
11. Zamárraga, M, Bigi F, Alito A, Romano MI, Cataldi AA (1999) A 12.7 kb fragment of the *Mycobacterium tuberculosis* genome is not present in *Mycobacterium bovis*. Microbiology 145: 893-897.
12. Forbes BA, Hicks KE (1996) Substances interfering with direct detection of *Mycobacterium tuberculosis* in clinical specimens by PCR: effects of bovine serum albumin. J Clin Microbiol 34: 2125-2128.
13. Abu Al-Soud W, Rådström P (2000) Effects of amplification facilitators on diagnostic PCR in the presence of blood, feces and meat. J Clin Microbiol 38: 4463-4470.
14. Kamerbeek J, Schouls L, Kolk A., van Agterveld M, van Soolingen D et al (1997) Simultaneous detection and strain identification of *Mycobacterium tuberculosis* for diagnosis and epidemiology. J Clin Microbiol 35: 907-914.
15. Viana-Niero C, Gutierrez C, Sola C, Filliol I, Vincent V et al. (2001) Genetic diversity of *Mycobacterium africanum* clinical isolates based on IS*6110*-restriction fragment length polymorphism analysis, spoligotyping and variable number of tandem DNA repeats. J Clin Microbiol 39: 57-65.
