# Supplementary material for: Detection and Molecular Characterization of 9000-Year-Old Mycobacterium tuberculosis from a Neolithic Settlement in the Eastern Mediterranean
Source: PLoS One. 2008 Oct 15;3(10):e3426. doi: 10.1371/journal.pone.0003426 (PMC2565837; doi:10.1371/journal.pone.0003426)
Supplement: Table S1 — The solvent sequence used for the silica gel normal phase cartridge fractionation of long-chain compounds (0.03 MB DOC) [file pone.0003426.s002.doc]

**Table S1**. The solvent sequence used for the silica gel normal phase cartridge fractionation of long-chain compounds.

| Fraction | Heptane % | Toluene % | CHCl_3_ % | Methanol % | Total ml |
| --- | --- | --- | --- | --- | --- |
| 1 | 100 |  |  |  | 6 |
| 2 | 97 | 3 |  |  | 6 |
| 3 | 90 | 10 |  |  | 6 |
| 4 | 80 | 20 |  |  | 6 |
| 5 | 50 | 50 |  |  | 6 |
| 6 |  | 100 |  |  | 6 |
| 7 |  |  | 100 |  | 6 |
| 8 |  |  | 80 | 20 | 6 |
| 9 |  |  | 50 | 50 | 6 |
