# Supplementary material for: Detection and Molecular Characterization of 9000-Year-Old Mycobacterium tuberculosis from a Neolithic Settlement in the Eastern Mediterranean
Source: PLoS One. 2008 Oct 15;3(10):e3426. doi: 10.1371/journal.pone.0003426 (PMC2565837; doi:10.1371/journal.pone.0003426)
Supplement: Table S2 — The solvent sequence used for the reverse phase cartridge purification of PBA-PFB mycolates (0.02 MB DOC) [file pone.0003426.s003.doc]

**Table S2**. The solvent sequence used for the reverse phase cartridge purification of PBA-PFB mycolates.

| Fraction | Acetonitrile% | Dichloromethane% | Total ml |
| --- | --- | --- | --- |
| 1 | 100 | 0 | 6 |
| 2 | 90 | 10 | 6 |
| 3 | 80 | 20 | 6 |
| 4 | 75 | 25 | 6 |
| 5 | 70 | 30 | 6 |
| 6 | 50 | 50 | 6 |
| 7 | 30 | 70 | 6 |
| 8 | 0 | 100 | 6 |
