# Supplementary material for: Detection and Molecular Characterization of 9000-Year-Old Mycobacterium tuberculosis from a Neolithic Settlement in the Eastern Mediterranean
Source: PLoS One. 2008 Oct 15;3(10):e3426. doi: 10.1371/journal.pone.0003426 (PMC2565837; doi:10.1371/journal.pone.0003426)
Supplement: Table S3 — Conditions for HPLC analysis of PBA-PFB mycolates (0.02 MB DOC) [file pone.0003426.s004.doc]

**Table S3**. Conditions for HPLC analysis of PBA-PFB mycolates

| Conditions | Column | Gradient elution program |
| --- | --- | --- |
| Flow rate: 1ml/min  Detector: Excitation 342nm, emission 376nm | Reverse phase | Acetonitrile/tetrahydrofuran: 55:45 to 45:55 in 31min |
|  | Normal phase | Heptane/ethyl acetate: 100:0 to 99:1 in 1min; 99:1 to 97:3 in 30min |
