# Supplementary material for: Detection and Molecular Characterization of 9000-Year-Old Mycobacterium tuberculosis from a Neolithic Settlement in the Eastern Mediterranean
Source: PLoS One. 2008 Oct 15;3(10):e3426. doi: 10.1371/journal.pone.0003426 (PMC2565837; doi:10.1371/journal.pone.0003426)
Supplement: Table S4 — Percentage ratios of alpha-, methoxy- and ketomycolates in archaeological samples and M. tuberculosis standard determined in normal phase HPLC (0.02 MB DOC) [file pone.0003426.s005.doc]

**Table S4**. Percentage ratios of alpha-, methoxy- and ketomycolates in archaeological samples and *M. tuberculosis* standard determined in normal phase HPLC

| **Mycolate** | **Woman left rib** | **Woman right rib** | **Infant** | ***M. tuberculosis* standard** |
| --- | --- | --- | --- | --- |
| **Alpha-** | 56.3 | 52.65 | 53.2 | 48.2 |
| **Methoxy-** | 25.2 | 34.50 | 32.2 | 37.4 |
| **Keto-** | 18.5 | 12.85 | 14.6 | 14.4 |
