# Supplementary material for: Detection and Molecular Characterization of 9000-Year-Old Mycobacterium tuberculosis from a Neolithic Settlement in the Eastern Mediterranean
Source: PLoS One. 2008 Oct 15;3(10):e3426. doi: 10.1371/journal.pone.0003426 (PMC2565837; doi:10.1371/journal.pone.0003426)
Supplement: Table S5 — Absolute amounts of mycolic acids extracted from bone samples (0.02 MB DOC) [file pone.0003426.s006.doc]

**Table S5**. Absolute amounts of mycolic acids extracted from bone samples.

| **Bone samples** | **Bone mass** | **PBA PFB mycolate in bone samples (µg)** | **Mycolate pg/ bone mg** |
| --- | --- | --- | --- |
| **Woman left rib** | 635 mg | 12.800 µg | 20.14 pg/mg |
| **Woman right rib** | 483 mg | 1.697 µg | 3.51 pg/mg |
| **Infant** | 589 mg | 0.073 µg | 0.12 pg/mg |
