# Supplementary figures and images for: Detection and Molecular Characterization of 9000-Year-Old Mycobacterium tuberculosis from a Neolithic Settlement in the Eastern Mediterranean
Source: PLoS One. 2008 Oct 15;3(10):e3426. doi: 10.1371/journal.pone.0003426 (PMC2565837; doi:10.1371/journal.pone.0003426)

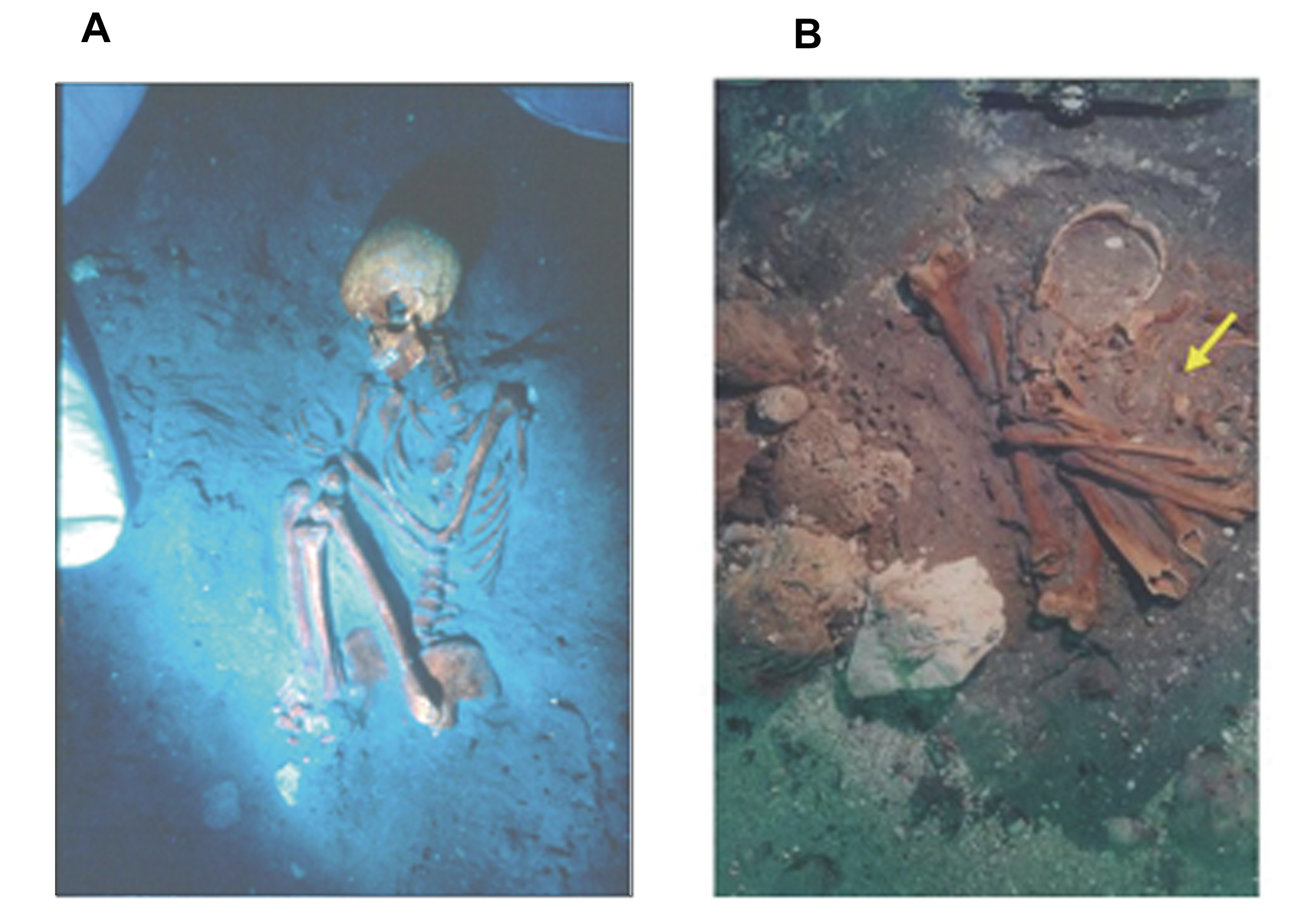

Supplement: Figure S1 — Atlit-Yam burials. A. An example of human remains with excellent preservation. B. Partial excavation of the burial site with the adult female and infant skeleton (arrow). (2.46 MB TIF) [file pone.0003426.s007.tif]

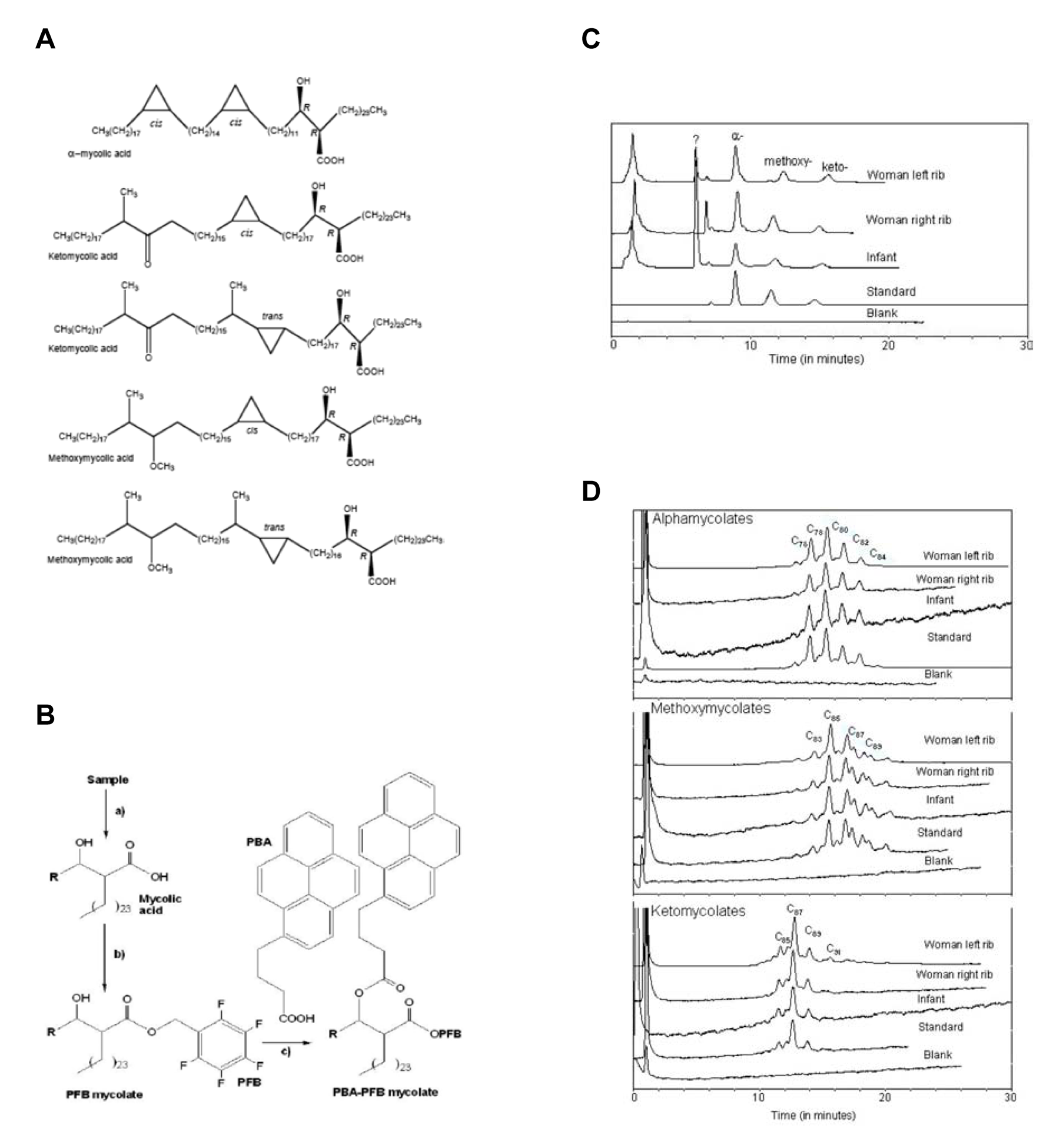

Supplement: Figure S2 — High Performance Liquid Chromatography (HPLC) methodology. A. Representative structures of the mycolic acids from M. tuberculosis. Natural mixtures of mycolates express a range of homologous components with varying chain lengths. B. Strategy for the release and derivatization of mycolic acids for fluorescence HPLC. (a) Hydrolysis with KOH/ methanol/toluene to release mycolic acids. (b) Phase-transfer catalyzed esterification of mycolic acids with pentafluorobenzyl bromide (PFB) to give PFB mycolates. (c) Esterification of PFB mycolates, by reaction with pyrenebutyric acid (PBA), to produce PBA-PFB mycolates, catalyzed by dicyclohexylcarbodiimide and pyrrolidinopyridine. R- represents the remainder of the mycolate molecule. C. Normal phase HPLC of PBA-PFB mycolates from bone samples and standard M. tuberculosis. D. Reverse phase HPLC of individual α -, methoxy- and ketomycolic acid PBA-PFB derivatives from bone samples and standard M. tuberculosis. The number of carbons in the individual underivatized mycolic acids is shown. (0.83 MB TIF) [file pone.0003426.s008.tif]

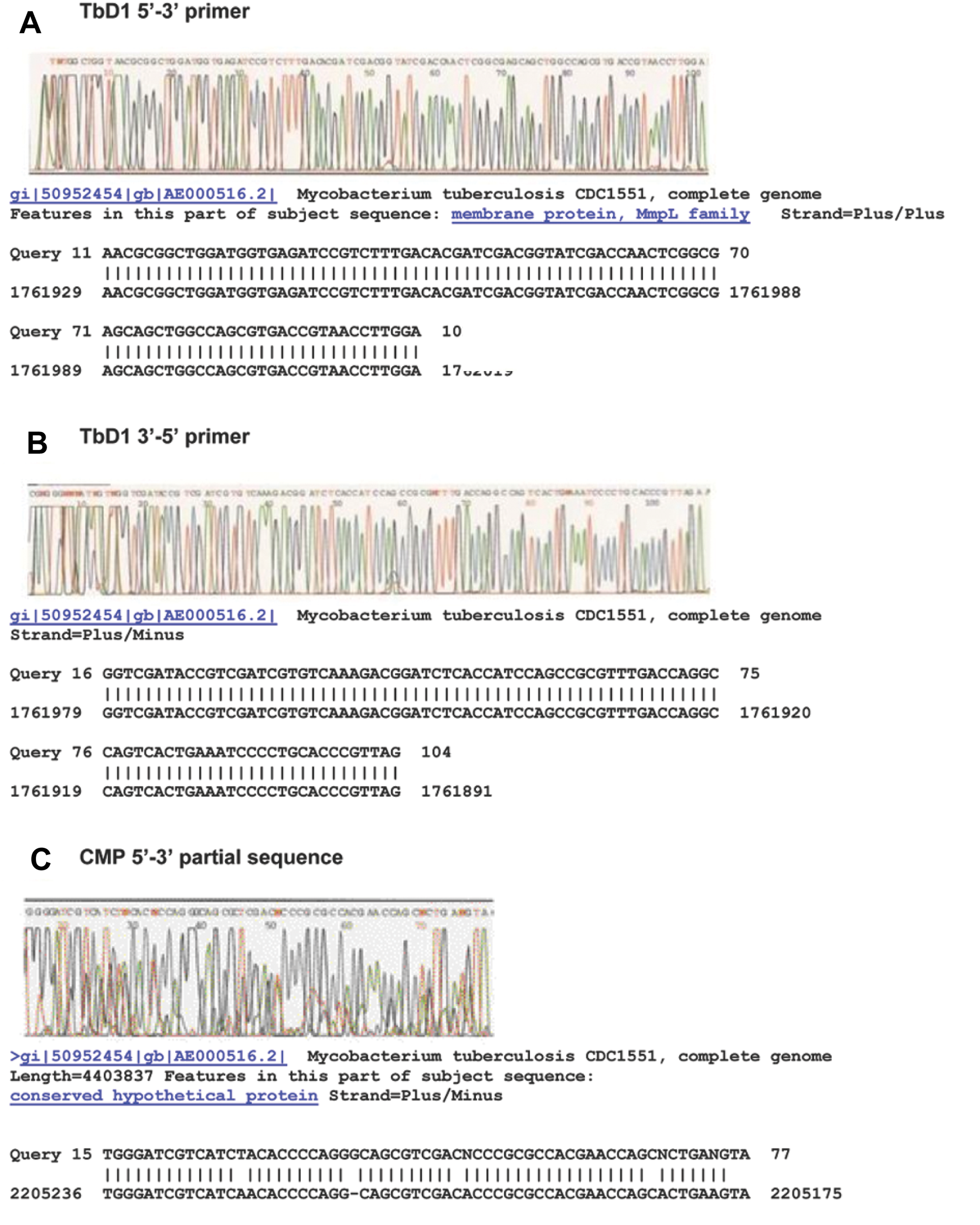

Supplement: Figure S3 — DNA sequence data. A. M. tuberculosis TbD1 flanking region (128 bp), obtained from the infant sample (5′-3′ strand). B. M. tuberculosis TbD1 flanking region (128 bp), obtained from the infant sample (3′-5′ strand). C. M. tuberculosis conserved membrane protein (CMP) region, obtained from the infant sample (5′-3′ strand only). (12.09 MB TIF) [file pone.0003426.s009.tif]

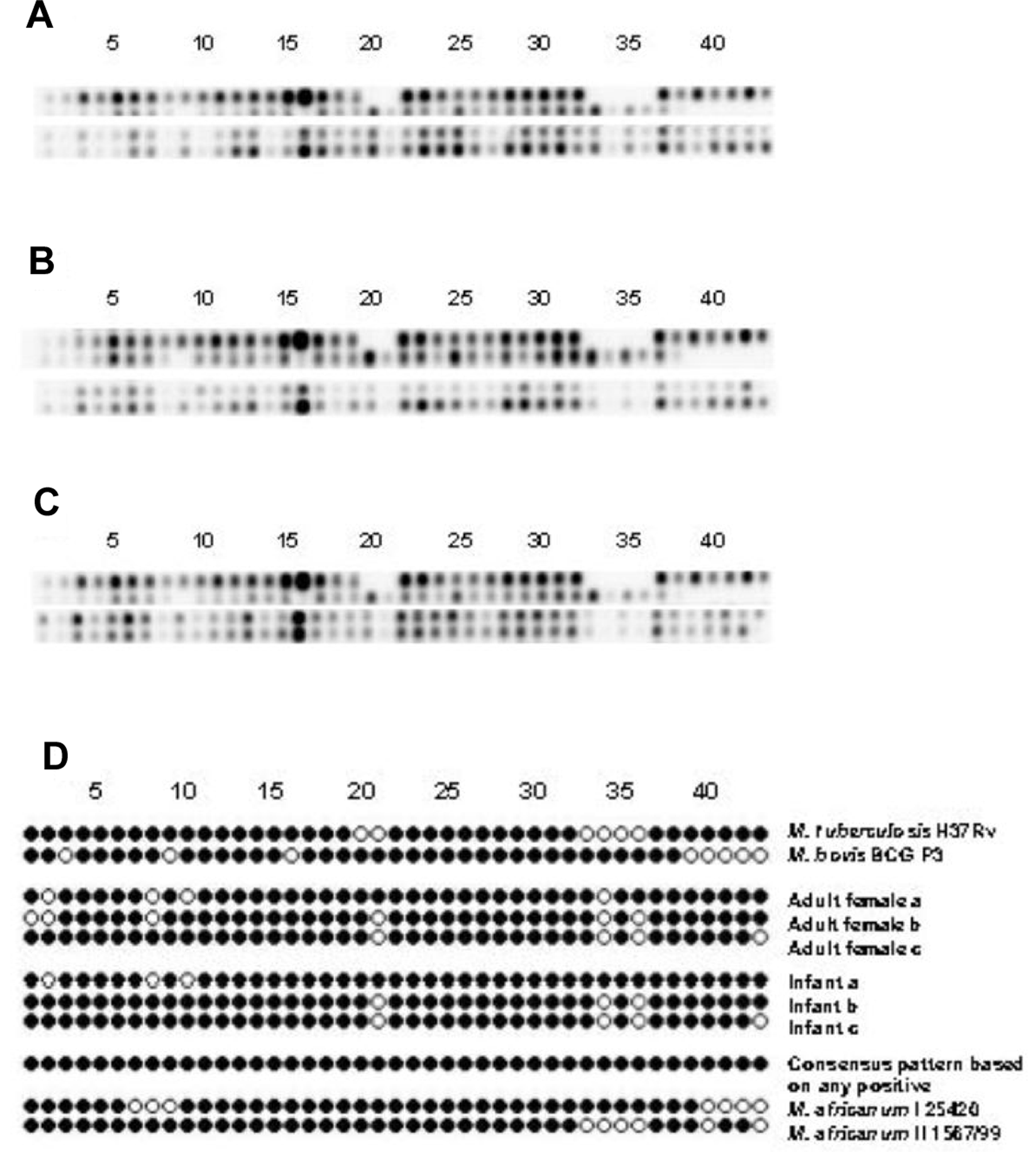

Supplement: Figure S4 — Repeated spoligotypes from the Atlit-Yam samples and controls. Each set of spoligotyping data (A–C) represents, from top to bottom, M. tuberculosis, M. bovis (BCG) controls, Atlit Yam female and Atlit Yam infant. D. Diagram of spoligotyping data, including M. africanum spoligotypes (Donoghue et al 2004), and a consensus pattern based on one or more positive results. (5.61 MB TIF) [file pone.0003426.s010.tif]
